# Lentigo Maligna Treatment—An Update

**DOI:** 10.3390/jcm13092527

**Published:** 2024-04-25

**Authors:** Loredana Ungureanu, Alina Florentina Vasilovici, Ioana Irina Trufin, Adina Patricia Apostu, Salomea-Ruth Halmágyi

**Affiliations:** 1 Department of Dermatology, “Iuliu Hațieganu” University of Medicine and Pharmacy, 400006 Cluj-Napoca, Romania; 2 Department of Dermatology, Emergency County Hospital, 400006 Cluj-Napoca, Romania; 3 Clinical Hospital of Infectious Diseases, 400000 Cluj-Napoca, Romania

**Keywords:** lentigo maligna, treatment, surgery, imiquimod, radiotherapy

## Abstract

Lentigo maligna (LM) is a melanoma in situ that is prevalent in chronically sun-damaged skin. Characterized by a slow growth pattern and high mutation rates due to chronic UV exposure, LM poses diagnostic and therapeutic challenges, particularly given its tendency to mimic other skin lesions and its occurrence in cosmetically sensitive areas. Its diagnosis is based on an integrated approach using dermoscopy and reflectance confocal microscopy (RCM). Despite its slow progression, LM can evolve into lentigo maligna melanoma (LMM), making its treatment necessary. Treatment modalities encompass both surgical and non-surgical methods. Surgical treatments like Wide Local Excision (WLE) and Mohs Micrographic Surgery (MMS) aim for clear histological margins. WLE, a standard melanoma surgery, faces challenges from LM’s subclinical extensions, which increase the recurrence risk. MMS, effective for large or poorly defined lesions, is defined by precise margin control while considering cosmetic outcomes. Non-surgical options, including radiotherapy and imiquimod, are alternatives for non-surgical candidates. Radiotherapy has been effective since the 1950s, offering good control and cosmetic results, especially for older patients. Imiquimod, an immunomodulator, shows promise in treating LM, though its application remains off-label. The increasing incidence of LM/LMM necessitates a balance in treatment choices to minimize recurrence and maintain cosmetic integrity. A multidisciplinary approach, integrating clinical examination with dermoscopy and RCM and histological assessment, is essential for accurate diagnosis and effective LM management.

## 1. Introduction

Lentigo maligna (LM) is a subtype of melanoma in situ (MIS) arising on chronically sun-damaged skin, comprising 4–15% of all melanomas, and 79–83% of all MIS cases [1,2]. LM is a slowly growing MIS, exhibiting a long radial growth pattern before invading the dermis and becoming lentigo maligna melanoma (LMM) [1,2,3]. LMM has the same prognosis as other types of invasive melanomas, influenced by Breslow thickness, ulceration, and other histological and clinical features [3]. The reported risk of progression varies in different studies between 2% and 20%, with the real risk being difficult to ascertain [3].

The incidence of melanoma arising on chronically sun-damaged skin is difficult to estimate, but evidence suggests that LM and LMM rates are steadily increasing, faster than any other melanoma subtype [2]. Because of the association with cumulative sun damage, LM/LMM is much more common in elderly people; however, a multicentric study conducted by Longo et al. showed that LM is not a disease limited to the elderly [4]. LM presenting in young adults tends to be smaller and with fewer dermoscopic criteria, which makes the diagnosis difficult [4].

Regarding pathogenesis, LM is linked to cumulative skin damage induced by chronic exposure to UV radiation, being located especially on the skin of the head and neck, or more rarely on the upper trunk and extremities, mainly the arms [3,5]. LM is associated with solar lentigines, a personal history of skin cancer, multiple actinic keratoses, fair skin, and to a lesser extent with melanocytic nevi and sun burns [3]. LM has one of the highest mutation rates of all human cancers due to chronic UV exposure, which decreases immune surveillance and leads to immunosuppression [2]. LM presents as a macule or patch with multiple colours, ill-defined edges and irregular borders located on chronically sun-exposed areas. Although LM arises usually on sun-damaged skin, being surrounded by multiple freckles, it can also present as a solitary facial macule in younger age [6]. Clinically, LM can be difficult to differentiate from its mimickers, including pigmented actinic keratosis (PAK), solar lentigines (SL), seborrheic keratosis (SK) and lichen planus-like keratosis (LPLK) [1,2,3,4,5,6]. Dermoscopy and reflectance confocal microscopy (RCM) increase the sensitivity and specificity of LM diagnosis compared with the naked eye examination alone [1]. Dermoscopic features of LM include the presence of grey dots, grey circle/semicircles, target-like patterns, including a circle within a circle, angulated lines, rhomboid structures, obliterated follicles, irregular hyperpigmented areas, irregular blotches, shiny white streaks, atypical vessels and erased areas [7]. Some of the above structures can also be seen in LM mimickers, so an inverse dermoscopic approach has been proposed to increase the diagnostic accuracy. This approach involves identifying and ruling out the characteristics of benign lesions, rather than focusing on malignant features [8]. RCM, an in vivo non-invasive technique that allows the evaluation of skin lesions at cellular resolution, allows a sensitivity of 93% and a specificity of 76% in the diagnosis of pigmented lesions [9]. RCM criteria for the diagnosis of LM/LMM include atypical cells in different skin layers, folliculotropism, medusa head-like structures, sheet-like structures, bulging around the follicle, junctional or dermal nests, nucleated cells within the papillae and inflammation (melanophages) [7]. Optical coherence tomography (OCT) is a non-invasive imaging technique mostly used for non-melanoma skin cancers, as it is capable of creating vertical images down to the deep dermis, reconstructing 3D images, measuring tumor thickness, and even distinguishing between basal cell carcinoma subtypes. Unfortunately, melanocytic lesions cannot be clearly visualized with OCT due to its lack of cellular resolution [10,11]. Line-field confocal optical coherence tomography (LC-OCT) is a new non-invasive system that offers a high penetration depth like OCT and similar high resolution to RCM for real-time visualization of images in both horizontal and vertical views [12,13]. In a case report published by Verzi et al., LC-OCT detected in vivo the main histological characteristics of LM: the presence of large, bright, roundish, or dendritic atypical cells with clear nuclei in the epidermis and around the hair follicles, corresponding to atypical melanocytes with a tendency toward folliculotropism. In the case of LM, LC-OCT has the great advantage of providing vertical images that allow for a more accurate evaluation of the dermo-epidermal junction, which plays a decisive role in dermal invasion [14]. In the future, LC-OCT may play a crucial role in the diagnosis of melanocytic lesions, potentially reducing the number of unnecessary biopsies, but there is need for further studies with clinicians of different experiences with LC-OCT and a larger number of patients [13,14].

The European consensus-based interdisciplinary guideline for melanoma recommends that the whole lesion should be completely excised, with a narrow margin of 1–3 mm, in order to perform histopathology when melanoma is suspected [15]. However, in large head and neck lentigo maligna, incisional biopsies may be necessary, including a punch, shave or eliptical/fusiform incisional biopsy [15]. An expert consensus agreed that obtaining multiple punch biopsies or a broad shave biopsy minimizes the risk of underestimation of LM, and recommended that the area to be biopsied should be chosen based on dermoscopy or RCM [1]. In LM, histopathological examination reveals an increased density of atypical melanocytes along the dermoepidermal junction with frequent adnexal involvement [1]. The same expert consensus agreed that LM diagnosis should be based on a clinical, dermoscopical, RCM and histological integrated approach [1].

Although slow growing, LM can sometimes progress to an invasive, potentially fatal melanoma, so treatment is necessary. The purpose of treatment is to completely remove the lesion with maximum preservation of normal tissue to minimize the risk of recurrences and to avoid functional and cosmetic deformities. However, selecting the best therapeutic approach can be challenging due to multiple reasons. Firstly, LM tends to occur on important cosmetic areas, making correct surgical treatment difficult. Moreover, LM margins are frequently ill defined, and the presence of subclinical extensions makes it difficult to define safe surgical margins. Non-surgical interventions can be an alternative in case of surgical ineligibility [1,2,3,4,5,6,7,8,9,15].

The present review aims to present an overview of LM treatment. LM can be treated using different approaches, including surgery, radiotherapy, blind destructive procedures (laser, cryosurgery), and topical therapies (imiquimod, 5-FU). A systematic review of surgical and non-surgical treatment options for MIS, including LM, failed to find high-quality evidence supporting the effectiveness of any of the treatments [16]. However, surgical excision to achieve clear histological margins is the first-line treatment according to guidelines [15,17]. As the incidence of LM/LMM is steadily increasing, it is crucial for dermatologists to select successful treatment options associated with a low risk of recurrence and acceptable cosmetic outcome.

## 2. Surgical Treatment

Guidelines recommend surgical excision as first-line treatment in LM, as previously mentioned [15,17]. This allows full histologic examination, being able to detect a potential invasive component. Studies demonstrate that a significant proportion of lesions (16–32.5%) classified as non-invasive during partial biopsy are upstaged to invasive tumor after complete excision [2].

### 2.1. Wide Local Excision

Wide local excision (WLE) with immediate closure of the defect is the standard surgical technique in melanoma surgery, including LM. Since 1992, the National Institutes of Health (NIH) established that there should be a 5 mm excisional safety margin for MIS, including LM [6]. However, surgical treatment of LM is associated with an increased risk of local recurrence compared with other subtypes of melanoma, as a result of incomplete resection. Unexpected positive margins are caused by the frequent subclinical extension of atypical cells [18]. Following WLE with the recommended 5 mm surgical margins, studies report recurrence rates that vary from 6% to 20%. A systematic review and meta-analysis on surgical techniques in LM showed that a 5 mm surgical margin resulted in negative histological margins in 83% of cases following WLE or staged excision with partial margin assessment, while after complete histological assessment, the margins were found to be involved in 59% of cases. The weighted mean of a surgical margin needed for a negative histological margin was 7.7 ± 2.00 mm [18]. As a consequence, newer guidelines acknowledge the need to use clinically measured surgical margins larger than 5 mm (i.e., 5–10 mm) in order to achieve local control. However, these safety margins are not always feasible in LM due to cosmetic and/or functional considerations. Moreover, LM is histologically analyzed via vertical bread loaf, permanent sections after WLW, allowing the examination of less than 1% of the specimen surface area, leading to an uncertain pathological clearance of the margins and an unpredictable risk of recurrence [19,20,21]. Permanent sectioning of the specimen leads to a delay between excision and the final pathological report, and in the presence of a positive margin, a second excision requiring another closure is needed [21].

In order to overcome these problems, several surgical techniques were developed for better assessing margins and detecting subclinical extension. These techniques include Mohs micrographic surgery (MMS) and staged excision procedure.

### 2.2. Mohs Micrographic Surgery

MMS is a specialized form of skin surgery that allows complete histopathological analysis of the peripheral and deep surgical margins; it minimizes tissue removal and spares normal tissue, while also being associated with the lowest rate of recurrence [22]. In LM, the initial stage consists of marking and surgically debulking the clinically evident tumor. According to guidelines, the debulked specimen has to be sent for permanent vertical sectioning in order to rule out invasive disease [19,20,21,22]. In a second step, an additional peripheral and deep margin of the tissue in a bowl-like configuration is taken, and flattened to ensure that the peripheral and deep margins are on the same plane, and immediately frozen in a cryostat. The sections cut from the flattened tissue are stained and examined by the surgeon. The technique allows for complete margin assessment, and if a residual tumor is noted, the process is repeated until the margins are histologically clear in which a new margin around the positive area is removed, processed and assessed for a residual tumor [22]. If invasive disease is found, permanent section analysis of the tissue block is recommended for correct staging [21].

MMS is recommended especially for large and/or poorly defined LM and for lesions located in anatomically difficult areas, such as the face, the ears, and the scalp [21]. The main advantages are complete marginal analysis and tissue preservation, while the disadvantages are represented by the risk of misinterpretation of true depth of invasive disease when found due to oblique tissue orientation, and the difficulty of discriminating between melanocytic hyperplasia of sun-damaged skin and tumor cells in frozen sections, particularly at the periphery of the tumor [21].

In order to assist in intraoperative margin control during MMS excision, immunohistochemical staining able to be implemented on frozen sections can be used. Such stains include Mel-5, S-100, HMB-45, and MART-1 [19]. Although melanocytes are highlighted by additional immunostaining, one study revealed that only 22% of all Mohs surgeons use them in practice due to the lack of knowledge, increased costs, and prolonged tissue processing time [23]. On the other hand, larger margins were reported by Kunishige et al. when using MART-1, emphasizing the fact that atypical cells may be overlooked on initial H&E staining, and more likely to be identified with MART-1 staining, leading to an overall larger surgical defect [24]. Elshot et al. conducted a meta-analysis on the surgical management of LM/LMM and showed that the use of MMS combined with IHC exhibited the lowest local recurrence rate [18]. Moreover, there is no consensus on the optimal use of immunostaining [18].

A significant controversy in MMS for LM is represented by the recommended margins for excision. Recently, Kunishige et al. conducted a large study in order to clarify the appropriate margin and concluded that for lesions on the trunk and extremities, a 9 mm margin should be used, while for lesions on the head and neck, 12 mm margins are recommended [24]. Interestingly, in their study, only 79.4% of LM tumors were completely excised when 6 mm margins were sampled [24].

### 2.3. Modified Mohs Surgery

A cylindrical excision down to the fascia or deep fat is used in modified MMS, both for the central debulk and the additional margin [21]. As for standard MMS, if a positive margin is detected, another layer of tissue is removed and analyzed, and permanent sectional analysis should be performed if there is invasive disease detected on the margins. The advantage of modified MMS is that the margins are processed with vertical en face sectioning avoiding the oblique tissue orientation issue encountered in the case of traditional MMS [25]. The indications, advantages, controversies and disadvantages are similar to those reported for the traditional technique.

### 2.4. Staged Excision/Slow Mohs with the En Face Protocol

Staged excision, also referred to as slow Mohs, is similar to modified MMS, but permanent sectioning is used both on the central debulk and on the peripheral layers, the assessment is performed by a dermatopathologist within 24–48 h. In order to avoid delay and ensure accurate assessment, the pathologist should be trained in and familiar with the technique [21].

Staged excision has the same indications as MMS and offers optimal tissue conservation and margin control. Moreover, permanent sectioning is used, which is the gold standard histological evaluation according to melanoma guidelines. The use of permanent sectioning removes the need to takedown a repair when invasive disease is found [21]. As a disadvantage, surgery occurs in multiple sessions, usually over multiple days in case of repeated positive margins [21].

Multiple staged excision variants were described, including spaghetti, perimeter, geometric or square, mapped serial excision, and radial vertical techniques. The standard principles are followed, with the variations being related to the order of tissue removal and the method used for reading the peripheral margin [21].

A Delphi consensus among international experts on the diagnosis, management and surveillance for LM considers surgical excision the best option for LM treatment, and more specifically, surgery with controlled margins as the treatment of choice [1].

### 2.5. Surgical Margin Mapping for LM

Due to the high recurrence rate and location in cosmetically sensitive areas, non-invasive diagnostic techniques were tested in order to assist diagnosis and highlight LM margins. Clinical examination, Wood’s lamp examination and dermoscopic assessment showed limited accuracy [26]. In the last few years, reflectance confocal microscopy (RCM), a non-invasive imaging technique that proved to be more sensitive and specific than dermoscopy in the diagnosis of LM, was studied in detecting the margins and reducing the risk of positive margins. Studies showed that RCM reduces the rate of positive histological margins, reduces the number of necessary stages, leading to less local recurrences [18,26,27]. When margin-controlled surgery is not available, RCM is associated with a better demarcation of the lesion, avoiding incomplete excision. Moreover, even when margin-controlled surgery is available, the use of RCM leads to more precise and efficient surgical management of the lesion [18,26,27].

Experts consider RCM to be a useful adjuvant tool for LM diagnosis and margin mapping, although its use is limited by the relatively scarce availability and the need for extensive training [1].

## 3. Non-Surgical Treatment

Complete surgical excision represents the standard treatment for LM according to guidelines and experts [1,15,17]. However, due to the location, size, advanced age of the patient or patient’s preference, surgery is not always an acceptable option. As a consequence, efforts have been made to develop efficient alternative therapies [28]. According to experts, when surgery is not feasible or not accepted by the patient, imiquimod 5% cream as a monotherapy represents the treatment of choice, while radiotherapy represents a possible alternative treatment, but other topical treatments have also been used with inconsistent results, including cryotherapy, laser therapy, photodynamic therapy, 5-fluorouracil and tazarotene [1].

### 3.1. Imiquimod

Melanoma, particularly LM, is an immunogenic tumor, and immune-modifying agents such as imiquimod can be used as a treatment option. Imiquimod is a synthetic drug belonging to the imidazoquinolone family, being an immune response modifier with antitumor and immunoregulatory activities, enhancing both the innate and acquired immune response. Imiquimod activates macrophages and antigen-presenting cells via Toll-like receptor 7 signaling, promoting an inflammatory state. An inflammatory cascade is then triggered, with the host’s immune system being able to recognize and destroy tumor cells [29,30,31].

Imiquimod is licensed for the treatment of actinic keratosis, superficial basal cell carcinoma and genital warts, with its use in LM being off-label. Although experts recommend it as the treatment of choice in LM for non-surgical candidates, there is a lack of high-quality evidence supporting the role of imiquimod, with its use being encouraged by case reports, retrospective studies and trials [1,29,30,31]. Moreover, imiquimod therapy is mentioned in several international guidelines, including the European Association of Dermato-oncology (EADO) and the American Academy of Dermatology (AAD) guidelines [15,17]. Imiquimod is proposed as an alternative to surgery in patients not eligible for surgery or radiotherapy in the EADO guideline, and considered a second line treatment option in primary or adjuvant setting by the AAD guideline. In a practical guide for the use of imiquimod, Guitera et al. considered it to be a treatment option in patients with multiple comorbidities or multiple recurrences, in patients with repetitive histologic positive margins and in young patients who have a cosmetic issue with surgery [31]. However, because of the higher lifetime risk of developing invasive disease compared to older patients, this option should be chosen with caution in young patients and close follow-up is mandatory.

#### 3.1.1. Imiquimod as Primary Therapy

In patients who are not eligible for surgery or radiotherapy, imiquimod 5% can be used as a primary treatment option according to the published literature and in the experts’ opinion [1,29,30,31]. Reported clearance rates vary depending on the study, but three systematic reviews that analyzed the published data reported similar histological clearance rates of approximately 76% [28,32,33]. Before starting the therapy, LM diagnosis should be histologically confirmed, and signs of early invasion should be assessed clinically, by dermoscopy and if possible using RCM. The same techniques are used to establish the margins of the lesion and to assess the subclinical extension [29,30,31].

Although there is no standardized regimen, the application of imiquimod with 1–2 cm margins for 5–7 days per week, over 12 weeks, leads to the best outcomes [29,30,31]. The objective of the treatment is to achieve visible inflammation for at least 10–11 weeks, with the intensity of the inflammatory response being directly associated with the clearance rate. Lallas et al. showed that a robust inflammatory response (grade III) is associated with 13-fold higher odds of complete clinical response [34]. If the inflammation is extremely severe, leading to unacceptable pain or ulcerations, imiquimod should be stopped for a few days and resumed at a lower dose 2–3 days per week. On the other hand, if the inflammatory response is low, different strategies can be used to enhance it, including increasing application frequency or treatment duration, using occlusion with a bandage or applying a topical retinoid [29,30,31]. Guitera et al. proposed an algorithm of dose escalation in case of a low inflammatory response, with the first assessment being performed 14 days after starting therapy, and the following ones every 7 days. The first step is to increase the application schedule to 7 days per week, with a bandage being next applied overnight with cream under occlusion, and finally, a topical retinoid cream should be applied locally every morning, continuing the protocol with imiquimod in the evening. Bandaging and topical retinoid application should be stopped once the inflammatory reaction is induced, trying to maintain inflammation without significant pain and ulcerations [31].

The long-term recurrence rate reported in the literature varies from 2.2% to 24.5% [28,32,33]. A recent study conducted by Seyed Jafari et al. reported a recurrence rate of 12.6%, 23.5%, and 25.7% at 3, 5, and 10 years, respectively, after a median follow up of 8 years [35]. The disease-free survival rate was 87.4%, 76.5%, and 74.3% at 3, 5, and 10 years, respectively. Chambers et al. reported that a history of failed excision, <60 applications of imiquimod, <5 applications per week and partial clinical clearance are factors associated with local recurrence [36]. In another study, Gautschi et al. reported that the risk of local recurrence was significantly associated with the number of total melanocytes, basal and suprabasal melanocytes, and pagetoid spreading melanocytes in the baseline biopsy specimen [37]. As mentioned before, the intensity of the inflammatory reaction is a strong predictor of therapeutic response. Papanikolaou et al. showed that LM persisted in 100% of patients who failed to develop an inflammatory reaction, in 57% of those with a mild reaction, but only in 27% of those with a brief reaction [38].

The reported risk of progression to LMM in LM treated primarily with imiquimod varies in different studies between 1.3% and 9% [30]. The reduced penetration of imiquimod leads to its inability to treat follicular extension, being a risk factor for progression [29,30,31]. On the other hand, a potential invasive component could be missed due to a sampling error associated with punch or shave biopsy leading to a histopathologic misdiagnosis. The unavailability of the entire lesion for histological examination and the possibility of missing an invasive component represents a matter of concern regarding LM treatment with imiquimod [35].

Due to the risk of recurrence and the risk of progression, LM lesions treated with imiquimod should be biopsied after treatment and followed up for many years, since recurrence can occur after several years [29,30,31]. Clinically, recurrence should be suspected if a brownish pigmentation is observed in the treated area, especially with perifolicular distribution [3]. In a study conducted by Micantonio et al., dermoscopy was associated with 80% sensitivity and 56% specificity in detecting treatment failure. Asymetric hyperpigmented follicular openings and isolated, very fine brown dots were the most important dermoscopic criteria associated with treatment failure [39]. Recent studies showed that RCM can be used for monitoring the response to therapy and to detect relapses [40,41]. Assessment of treatment response should be performed only six months after treatment completion, because early biopsies could show persistent disease [29,30,31].

In order to increase the inflammatory reaction, imiquimod can be combined with other topical agents. Topical retinoids are the most frequently used, with both tazarotene and tretinoin enhancing the penetration of imiquimod by disrupting the epidermal barrier. Hyde et al. showed that the combination of imiquimod with tazarotene 1% gel leads to a significant increase in clinical inflammatory response, but not a significant increase in the histological clearance rate [42]. Nahm et al. combined imiquimod with 5-fluorouracil 2% and tretinoin 0.1% and achieved complete histological clearance confirmed by biopsies 3 months after treatment completion in two cases. Moreover, 5-fluorouracil acts as an antimetabolite, leading potentially to synergic antitumor activity with imiquimod [43].

#### 3.1.2. Imiquimod as Adjuvant Therapy

According to melanoma guidelines, imiquimod can be used as adjuvant therapy in LM after surgery with histologically affected or negative but narrow margins [1,15,17].

High clinical and histological clearance rates, ranging between 93 and 95% in large series, have been reported for adjuvant therapy with imiquimod after surgery in LM. The long-term recurrence rate varies between 6.5 and 7%, a lower recurrence rate than the one reported following WLE, and closer to the one reported after surgery with microscopic margin control [30,34,44]. Positive surgical margins are significantly associated with decreased clearance rates as compared to narrow surgical margins, while the intensity of the inflammatory response during treatment is directly associated with the clearance rate [44].

The best regimen for adjuvant therapy consists of 5–7 applications per week, with at least 60 applications and a 2 cm margin of clinically normal skin, adjusting the application schedule to achieve an inflammatory reaction without significant pain or ulcerations. If necessary, topical retinoids may be added to reduce inflammation [30,34,44].

Some authors proposed the use of imiquimod as an adjuvant for laser ablative therapy (CO_2_ laser or erbium-doped yttrium aluminium garnet laser). An ablative laser was used with a 2–3 cm margin of clinically normal skin, followed by five applications per week of imiquimod 5% for 6 weeks [45,46]. A total of 35 LM patients were treated, with a mean follow-up of 19 months, and a recurrence rate of 9.4%, and 23.5% at 1, and 3 years, respectively, a lower recurrence rate than that reported for laser monotherapy. By removing the epidermis and superficial dermis, laser therapy eliminates the majority of malignant melanocytes, while also allowing a deeper penetration of imiquimod, with increased inflammation and an increased clearance rate [45,46].

#### 3.1.3. Imiquimod as Neoadjuvant Therapy

In the last few years, imiquimod was proposed as neoadjuvant therapy before surgical excision in order to reduce the size of the lesion by targeting the subclinical extension, and thus reducing the excision margins and required stages [47]. Different studies confirmed the hypothesis and showed that the off-label use of imiquimod leads to a smaller final surgical defect size associated with negative histological margins [42,47,48,49].

Donigan et al. used a protocol in which patients were treated with imiquimod 5% 5 days per week for 8–12 weeks, followed by staged excision, and reached a median final margin of 2 mm with a recurrence rate of 3.9% after a mean follow-up of 5.5 years, a recurrence rate similar to that reported following standard staged excision [48]. Sampson et al. firstly removed LM with excisional biopsy ruling out an invasive component; subsequently, they applied imiquimod 5 days per week for 8 weeks, and finally, they performed staged excision with 2 mm margins. They demonstrated that the protocol allows for a 71% reduction in the required margins compared with standard excision [49]. Using a similar protocol, but extending the imiquimod treatment to 12 weeks, Hyde et al. showed that LM margins previously treated with imiquimod are easier to interpret by the pathologist due to the clearance of atypical melanocytes associated with chronic sun exposure [42]. Flores et al. also demonstrated decreased melanocytic hyperplasia in LM treatment sites after five applications of imiquimod per week for a period of 8 weeks [50]. Daude et al. performed a prospective, randomized, multicenter, phase III clinical study in which patients were randomized to receive imiquimod or placebo for 4 weeks, followed by LM excision 4 weeks after the last application. Excision was performed using a 5 mm margin from the residual pigmentation after neoadjuvant therapy, and in the case of intralesional excision, a new excision with a 5 mm margin was performed before extralesional excision [47]. The study demonstrated that neoadjuvant treatment with imiquimod significantly reduces the LM area after 1 month of treatment, without being associated with a higher risk of intralesional excision [47].

All open-labelled studies evaluating neoadjuvant therapy with imiquimod demonstrated an extralesional excision rate ranging from 78 to 100% [42,47,48,49,50]. However, long-term follow-up using clinical-dermoscopic assessment, and also RCM if available, is mandatory in all cases treated with neo-adjuvant imiquimod.

### 3.2. Radiotherapy

Radiotherapy (RT) can be used for the treatment of LM either as primary treatment or as adjuvant therapy in the case of positive margins following excision [6,28,51,52]. RT was introduced into the treatment of LM in the 1950s by Miescher et al. [53]. The aim of this type of therapy is local control, avoidance of progression to invasive disease, and avoidance of radiation side effects so that the quality of life of the patient improves after therapy [51]. Several factors should be considered when choosing radiotherapy, including patient characteristics (performance status, life expectancy, ability to attend therapy), and tumor features (size, site, proximity to radiosensitive structures) [52].

A recent systematic review regarding radiotherapy for LM and LMM stated that the available evidence suggests that RT may be a safe and effective treatment for LM and LMM [51]. Grenz ray therapy and superficial radiotherapy are the two techniques that can be used. Grenz rays have a very low tissue penetration rate with a half-dose depth of about 1 mm, while superficial X-rays have a half-dose depth of more than one centimeter. A total dose of 100 Gy can be safely performed in Grenz ray RT, with a single fraction dose of 10–20 Gy, while in superficial RT, smaller single fractions of 2.0–7 Gy and a lower total dose are recommended (54–60 Gy) [51]. According to the same systematic review, the radiation field should include the visible lesion and a safety margin of at least 10 mm of the surrounding skin, in order to reduce the risk of out-of-field recurrences. Pre-treatment mapping biopsies or RCM mapping may help assess the subclinical extent of the lesion [52]. Although Grenz ray RT has been shown to achieve high local control rates of more than 90% in experienced centers, this technique was abandoned in the US due to the recurrence rate, presumably as a result of insufficient penetration into the skin appendages [51]. Fogarty et al. recommend favoring superficial RT or at least higher Grenz ray energies in order to completely cover the target volume with the therapeutic dose [52]. Moreover, the use of superficial X-rays is associated with lower dose inhomogeneities in concave or convex surface skin areas [51]. Overall, excellent complete response rates of 87–100% and recurrence rates varying from 0 to 31% were reported in studies using Grenz rays or superficial X-rays, comparable to recurrence rates after surgical excision and more favorable than following other non-invasive types of treatment [28,51,52].

The cosmetic outcome was reported as “good” to “excellent” in most cases, with the main side effects being the flattening of the epidermis, fibrosis, telangiectasias, pigmentary changes and atrophia [6,28,51,52]. The risk of radiotherapy-induced second cancers seems to be negligible in this elderly population [51].

Overall, RT seems to be associated with excellent local control, good cosmetic outcome and lower recurrence rates compared with other non-surgical treatments, being a good treatment option especially for elderly patients with lesions located in cosmetically sensitive areas that have contraindications or risk factors for surgery [28,51,52].

### 3.3. Cryosurgery

Although melanocytes are sensitive to damage induced by freezing temperature of −4 °C to −7 °C, there is no evidence that cryotherapy kills malignant melanocytes [6]. Moreover, the freezing temperature must penetrate deeply into the skin, including hair follicles, so the treatment should be aggressive, implying a double freeze–thaw cycle, with a freezing duration of 45 to 60 s required to freeze a lateral margin of apparently healthy skin of 1 cm, which is repeated after a thaw period of 2–3 min [6]. The reported rate of success is variable, but there are only a few studies and no established protocol available so far. Collins et al. [54] reported a recurrence rate of 40%, while Kuflick et al. [55] reported a 3-year recurrence rate of 6.6%.

Cryosurgery can be combined with imiquimod in the treatment for LM, thus increasing the inflammatory response, and consequently the rate of complete response. Many regimens have been described with different results, but there is no standardized treatment, cryosurgery being performed before, during or after imiquimod therapy [30].

### 3.4. Laser Therapy

Different types of laser therapy have been studied for the management of LM: Q-switched NdYag (neodymium: yttrium aluminum garnet), alexandrite, carbon dioxide, Er:YAG, or a combination of those. Early reports suggested good results, with superior cosmetic outcomes and good tolerability. However, further studies showed poor complete response rates and high recurrence rates with the possible explanation that some nests of malignant melanocytes remain untreated by the laser [28].

Orten et al. treated eight patients with a Q-switched neodymium: yttrium aluminum garnet laser and reported that all patients showed a response to laser therapy, including two patients demonstrating complete eradication of the LM, with no evidence of recurrence in 42 months [56].

After treating four patients with a carbon dioxide laser, Kopera et al. reported no recurrence after a mean follow-up of 15 months [57].

Madan et al. used QS:NdYAG and alexandrite lasers to treat histologically proven LM in 22 patients, with a follow-up period of 5 years. Complete clinical response was achieved in 12 patients, LMM developed in two lesions and recurrence occurred in 4 patients [58].

Lee et al. compared surgical excision, radiotherapy and carbon dioxide laser ablation on a cohort of 73 patients, with 15 receiving laser therapy. The reported recurrence rates were 4.2% for surgical excision, 29% for radiotherapy and 6.7% for carbon dioxide laser therapy [59].

In their study, Greveling et al. followed 35 patients for a median follow-up of 19 months after undergoing combination therapy with an ablative laser and imiquimod. They described a recurrence rate of 23.5%, with most of the recurrences being located on the nose [46]. The authors tried to determine histological parameters that might be related to the observed higher recurrences on the nose after non-surgical treatment and found that a higher density of atypical melanocytes and also a deeper extension into the follicles could be the explanation [46].

Fikrle et al. reported the use of a 2940 nm Er:YAG (erbium-doped yttrium aluminum) laser on 17 patients with a histopathologically confirmed lentigo maligna. The lesions were treated with a 5 mm margin of adjacent skin under local anesthesia. The lesion was retreated during the following three months if clinically visible pigmentation was observed in the ablated area and the patients were followed for residual or recurrent tumors. Complete clinical clearance with good cosmetic outcomes was achieved in all 17 patients, and three recurrences were detected during the follow-up period (9, 30 and 36 months after laser therapy) [60].

Kaur et al. reported the use of a CO_2_ laser with or without imiquimod for the treatment of 25 patients with 27 LM lesions. In total, 14 patients underwent combined therapy and 11 patients were treated with laser therapy alone, with a mean follow-up of 6.8 years. In addition, 18 patients (9 in combined therapy group and 9 in laser-only group) had clinical recurrence of pigment in the treated area, with histological confirmation being performed in only 6 patients. On average, recurrence was observed after 5.8 years, and the most common sites for recurrence were cheek and nose areas. The study showed high recurrence rates, especially after long-term follow-up, both for the laser-only and combined therapy group, although it was considered that topical imiquimod following laser therapy allows deeper penetration of the drug [61]. Zalaudek et al. evaluated the influence of sex, age, body site and treatment modalities on recurrence rates in MIS and found the highest recurrence rate after laser therapy (42.9%), compared with 6.8% for surgical excision, 34.3% for cryotherapy and 13.2% for radiotherapy [62]. In a recent review regarding non-surgical treatments for LM, Read et al. reported a mean recurrence rate of 34.4% after laser therapy, with a follow-up period of 8–78 months; the mean recurrence rate after radiotherapy and topical imiquimod was 11.5% and 24.5%, respectively [28].

Aiming to obtain better results, some authors thought to combine laser therapy with other treatment modalities. One study prospectively investigated the combination of an ablative fractional laser with photodynamic therapy (PDT), making deep channels with the CO_2_ laser in order for the photosensitizer to enter deeply enough into the skin and reach all the atypical melanocytes. They included ten histologically confirmed LMs that were treated with ablative fractional laser-assisted PDT with 5-aminolaevulinic acid nanoemulsion for three sessions at a 2-week interval with a total dose of 90 J/cm^2^ per session, and obtained a moderate efficacy with a complete histopathological clearance of 70% (7/10). The pathological report was obtained after surgical excision of the treated lesion, 4 weeks after the last laser-assisted PDF [63].

### 3.5. Photodynamic Therapy (PDT)

Based on the fact that photodynamic therapy with 5-aminolevulinic acid may determine in vitro lysis of melanocytes [64], there was one study conducted by Karam et al. that reported a cure rate of 80% (12/15) when treating LM with PDT with 5-aminolevulinic acid, but the efficacy of the treatment was histologically evaluated only partially by taking multiple biopsies [65].

### 3.6. Tazarotene

Tazarotene is a retinoid used for different inflammatory skin conditions like psoriasis, Darier’s disease, congenital ichthyosis. It has also been used in patients with basal cell carcinoma, with a good clinical response [66]. Small case series reported the use of this topical treatment in LM alone or in combination with imiquimod. Chimenti et al. described two elderly patients with LM treated with tazarotene gel 0.1% once daily for a period of 6 to 8 months with a complete response confirmed pathologically and with no recurrence after a follow-up period of 18 to 30 months [66]. Hyde et al. questioned whether topical retinoids can improve the complete response rate in patients with LM treated with imiquimod and conducted a study on ninety patients randomized in two groups: one treated with imiquimod 5% cream for 5 days/week for a period of 3 months, and the other one with the same schedule for imiquimod plus tazarotene gel 0.1% 2 days/week. Following topical therapy, all lesions underwent staged excision. More lesions treated with imiquiomd alone (36%) showed residual LM following staged excisions, compared to those treated with both imiquimod and tazarotene (22%) [42]. In a case report of LM, Menzies et al. observed no clinical improvement after treating the lesion with imiquimod alone for a total period of 14 weeks. Therefore, the authors added tazarotene gel once daily for a month, followed by imiquimod daily for 8 weeks and obtained clearance of the lesion, which was confirmed by multiple biopsies [67]. They emphasized that tazarotene enhanced the penetration of imiquimod. On the contrary, there are also studies suggesting that the addition of tazarotene to imiquimod may enhance the inflammatory response and cause the patients to stop the treatment [16].

### 3.7. 5-Fluorouracil (5-FU)

The use of 5-FU for treating LM was first mentioned in the literature in 1975, but there are few studies on this topic and this might be due to the negative results obtained [68]. All the patients treated topically with 5-FU presented recurrences [68,69].

## 4. Treatment of LM in the Real World

Several studies have reported variances in the management of LM among dermatologists. The latest was published by Tio et al. who conducted a survey in order to assess the diagnostic methods and clinical management of LM patients among European dermatologists and residents. They concluded that there is variance in the diagnostic and treatment modalities used for LM across Europe [70]. A combination of clinical diagnosis (65.7%), dermatoscopy (83.4%) and histopathology (88.2%) was reported by most respondents to diagnose LM. Regarding therapy, surgery remains the most frequently used option, both in younger and elderly patients, although the proportion is much higher in patients under 60 years of age compared with patients over 70 (97.6%, and 66.8%, respectively). Non-surgical options such as radiotherapy (17.0%), topical imiquimod (30.6%), watchful waiting (19.6%) or cryotherapy (20.4%) were used especially in the elderly populations. Interestingly, a sub-analysis showed that respondents who take into account patient preference used topical imiquimod, radiotherapy and watchful waiting to a larger extent [70].

## 5. Conclusions

LM is a subtype of MIS arising on chronically sun-damaged skin, with a constant growing incidence all over the world. Although LM is a slowly growing MIS, exhibiting a long radial growth pattern, it has the potential of invading the dermis and becoming LMM that has the same prognostic as other types of invasive melanomas. Therefore, treatment is necessary, with the aim to completely remove the lesion with maximum preservation of normal tissue, to minimize the risk of recurrences and to avoid functional and cosmetic deformities. Surgical excision to achieve clear histological margins is the first-line therapy according to guidelines, with surgery with controlled margins being the treatment of choice. When surgery is not possible or it is declined by the patient, different non-surgical options can be used. According to an expert consensus, imiquimod 5% cream as monotherapy represents the treatment of choice, while radiotherapy represents a possible alternative treatment. Life-long follow-up is mandatory, especially when using non-surgical treatments, due to the likelihood of late recurrences, but also in order to provide early detection of new skin tumors, either melanoma or keratinocyte carcinoma.

## Data Availability

No new data were created.
